# Effect of Intrinsic and Extrinsic Factors on the Pharmacokinetics of Antibody–Drug Conjugates (ADCs)

**DOI:** 10.3390/antib10040040

**Published:** 2021-10-15

**Authors:** Iftekhar Mahmood

**Affiliations:** Mahmood Clinical Pharmacology Consultancy, LLC., Rockville, MD 20850, USA; Iftekharmahmood@aol.com; Tel.: +1-301-838-4555

**Keywords:** antibody–drug conjugates, pharmacokinetics, intrinsic and extrinsic factors, monoclonal antibodies, small molecule or payload

## Abstract

Antibody–drug conjugates (ADCs) are complex molecules wherein a monoclonal antibody is linked to a biologically active drug (a small molecule), forming a conjugate. Initially, most of the ADCs were developed and are being developed for the treatment of cancer; however, with time, it has been realized that ADCs can also be developed to manage or cure other diseases. Pharmacokinetics (PK) plays an important role in modern-day drug development and the knowledge of PK is crucial in designing a safe and efficacious dose to treat a wide variety of diseases. There are several factors that can alter the PK of a drug; as a result, one has to adjust the dose in a patient population. These factors can be termed ‘intrinsic’ or ‘extrinsic’. For small molecules, the impact of both intrinsic and extrinsic factors is well established. The impact of age, gender, disease states such as renal and hepatic impairment, drug–drug interaction, food, and in many cases alcohol on the PK of small molecules are well known. On the other hand, for macromolecules, the impact of these factors is not well established. Since the ADCs are a combination product of a monoclonal antibody linked to a small molecule, both the small molecule and the monoclonal antibody of the ADCs may be subjected to many intrinsic and extrinsic factors. This review summarizes the impact of intrinsic and extrinsic factors on the PK of ADCs and the payloads.

## 1. Introduction

Monoclonal antibodies (mAbs) are widely used therapeutic agents to manage or cure a wide variety of diseases, especially in hematology and oncology. Although mAbs have shown therapeutic benefit in the field of oncology, these antibodies either do not have the optimal clinical efficacy or have to be co-administered with traditional chemotherapy. Therefore, in order to enhance the therapeutic benefit, there are efforts to enhance the efficacy of antibodies by forming a conjugate [1,2].

Antibody–drug conjugates (ADCs) are complex molecules wherein a monoclonal antibody is linked to a biologically active drug (a small molecule), forming a conjugate [1]. In 2000, the US FDA approved the first ADC Gemtuzumab ozogamicin (trade name: Mylotarg) for the treatment of CD33-positive acute myelogenous leukemi [1]. MYLOTARG was withdrawn from the market in 2010 due to adverse events, particularly hepatic side effects. It was then approved in 2017. Since the first approval of MYLOTARG in 2000, there has been enormous focus by the pharmaceutical companies to develop ADCs to treat a wide variety of diseases. Initially, most of the ADCs were developed and are being developed for the treatment of cancer; however, with time, it has been realized that ADCs can also be developed to manage or cure other diseases such as inflammatory diseases, atherosclerosis, and bacteremia [2]. The main objective of an ADC as a cancer agent is to release the cytotoxic drug to kill the tumor cells without causing any harm to the normal or healthy cells.

ADCs combine the selectivity of antibodies with the efficacy of small-molecule drugs, leading to more precise and targeted therapeutic applications. There are three components of ADCs: a mAb, a cytotoxic drug (small molecule drug also known as payload), and a specialized chemical linker which connects the mAb with the small molecule [3]. All these three components are very important in designing an ADC. The antibody portion of an ADC targets a specific antigen only found on target cells. Once it binds to the cell, it delivers the payload with a very high specificity to the diseased cells, maximizing their efficacy and minimizing systemic exposure. Payload is the crucial part of an ADC with cytotoxic capability. Payloads for ADCs can be small molecules, protein toxins, and peptides [3].

Since the ADCs are complex compounds, several analytes or moieties are found in the blood which can be detected by the available analytical methods. The analytes that are generally measured are: the conjugated antibody or ADC (antibody with drug), the total antibody (conjugated, partially de-conjugated, and fully de-conjugated), the antibody-conjugated drug (the small molecule drug conjugated to antibody), the un-conjugated drug (small molecule drug not conjugated to antibody), and possibly metabolites of the small-molecule drug including or not part of the linker [3].

In modern day drug development, pharmacokinetics (PK) plays an important role in designing a safe and efficacious dose to treat a wide variety of diseases. PK is a quantita-tive analysis of how living systems handle a molecule after its administration into a living organism. Pharmacokinetics is the study and characterization of the time course of drug absorption, distribution, metabolism, and excretion (ADME) [4]. The main objective of a PK study is to obtain information regarding ADME of a molecule [4]. These pharmacoki-netic parameters can then be used to design an optimal dosing regimen to facilitate Phase II and Phase III clinical trials [4]. The PK parameters can also be linked to the time course of pharmacological response (therapeutic and/or toxicologic) of a drug [5].

The important PK parameters of a molecule are the maximum plasma concentration (C_max_), area under the curve (AUC), clearance, elimination half-life, and volume of distribution. Generally, PK parameters of an ADC are determined for total antibody, ADC or conjugated mAb, and un-conjugated drug in blood or plasma [3]. ADCs are given by intravenous infusion. For a typical ADC and total antibody, C_max_ is reached at the end of the infusion and then the ADC concentrations decline mono- or multi-exponentially, with terminal half-lives ranging from 3 to 20 days. The total antibody concentrations in serum or plasma are higher than those of the ADC. The volume of distribution is either close to or slightly greater than the blood or plasma volume. The un-conjugated moiety follows a formation-limited kinetics and is at much lower concentrations than the total antibody or ADC [3,6]. Recently, a review leading to clinical pharmacology aspects of ADCs has been published [7].

## 2. Factors That Can Impact the PK of ADCs

There are several factors that can alter the PK of a drug; as a result, one has to adjust the dose in a patient population. These factors can be termed ‘intrinsic’ or ‘extrinsic’. Intrinsic factors are those related to an individual. For example, age, gender, genetics, and disease states are examples of intrinsic factors. On the other hand, extrinsic factors are the influence from outside. For example, concomitant medicine (drug–drug interaction), food or beverages (alcohol), smoking, malnutrition, water deprivation, and environment.

For small molecules, the impact of both the intrinsic and extrinsic factors are well established. The impact of age, gender, disease states such as renal and hepatic impairment, drug–drug interaction, food, and in many cases alcohol on the PK of small molecules are well known. On the other hand, for macromolecules, the impact of these factors is not well established, mainly because studies are lacking in this direction. Since the ADCs are a combination product of a monoclonal antibody linked to a small molecule, both the small molecule and the monoclonal antibody of the ADCs may be subjected to many intrinsic and extrinsic factors.

This review summarizes the impact of intrinsic and extrinsic factors on the PK of ADCs. This review mainly focuses on the approved ADCs by the United States Food and Drug Administration (US FDA) and most of the information were obtained from the FDA package inserts of the ADCs. Information was also obtained from the Assessment reports of the European Medicine Agency (EMA). This was done because published literature is scarce on the clinical pharmacology aspects of ADCs and the package inserts of the FDA and the assessment reports of the EMA are a good source of clinical pharmacology information. Following is the review of the impact of intrinsic and extrinsic factors on the PK of 10 ADCs approved by the FDA.

A brief description of 10 approved ADCs is presented in Table 1 [8,9,10,11,12,13,14,15,16,17]. The intrinsic and extrinsic factors discussed in this review are age (elderly (65 years and older) versus young), sex, race, pediatrics, renal and hepatic impairment, immunogenicity, and pregnancy and lactation. These factors are summarized in Table 2, Table 3, Table 4, Table 5 and Table 6 and are based on the FDA package inserts.

## 3. Age (Elderly (65 Years and Older) versus Young) 

The overall conclusion on age differentiating between elderly versus young was that there was not enough data (small sample size) to determine the difference in PK between these two age groups (*n* = 1), population PK (POPPK) could not detect the PK difference (*n* = 5), and no clinically meaningful (*n* = 4) PK difference between the two age groups was observed (Table 2).

**Table 2:** Impact of age, sex, and race on the pharmacokinetics of ADCs.

ADCs	Age (Young vs. Elderly)	Sex	Race	Pediatrics
ZYNLONTA (loncastuximab tesirine-lpyl), 2021	No clinically significant differences in the PK (20–94 years) was noted.	No clinically significant differences in the PK were noted.	No clinically significant differences in the PK (White and Blacks) were noted.	No systematic study was conducted.
BLENREP (belantamab mafodotin-blmf), 2020	No clinically significant differences in the PK were noted.	No clinically significant differences in the PK were noted.	No clinically significant differences in the PK were noted.	No systematic study was conducted.
TRODELVY (sacituzumab govitecan-hziy), 2020	POPPK did not detect any difference in the PK.	No information provided	POPPK did not detect any difference in the PK.	No systematic study was conducted.
ENHERTU (fam-trastuzumab deruxtecan-nxki), 2019	Population PK (POPPK) did not detect any difference in the PK.	POPPK did not detect any difference in the PK.	POPPK did not detect any difference in the PK.	No systematic study was conducted.
PADCEV (enfortumab vedotin-ejfv) 2019	No clinically significant differences in the PK were noted.	No clinically significant differences in the PK were noted.	No clinically significant differences in the PK were noted.	No systematic study was conducted.
POLIVY (polatuzumab vedotin-piiq) 2019	No clinically significant differences in the PK were noted.	No clinically significant differences in the PK were noted.	No clinically significant differences in the PK were noted.	No systematic study was conducted.
BESPONSA (inotuzumab ozogamicin), 2017	POPPK did not detect any difference in the PK (18–92 years).	POPPK did not detect any difference in the PK was noted.	POPPK did not detect any difference in the PK (Asian, non-Asian, Whites and Blacks).	No systematic study was conducted.
KADCYLA (ado-trastuzumab emtansine), 2013	POPPK did not detect any difference in the PK.	Most of the patients were females (breast cancer) hence, no evaluation on sex.	POPPK did not detect any difference in the PK.	No systematic study was conducted.
ADCETRIS (brentuximab vedotin), 2011	POPPK did not detect any difference in the PK. Clinical trials of ADCETRIS did not include sufficient numbers of patients aged 65 and over to determine whether the elderly respond differently from younger patients (<65 years).	POPPK did not detect any difference in the PK.	POPPK did not detect any difference in the PK.	A pediatric study was conducted. Please see the details of the pediatric study in the text [19].
MYLOTARG (gemtuzumab ozogamicin), 2000	No overall differences in effectiveness were observed between elderly and younger patients. Elderly patients experienced a higher rate of fever and severe or greater infections.	No clinically significant differences in the PK were noted.	No clinically significant differences in the PK were observed [20]. Please see the text.	A pediatric study was conducted. Please see the details of the pediatric study in the text [21].

## 4. Sex and Race

The overall conclusion was that sex did not have any clinical impact on PK (*n* = 5), POPPK could not detect the impact on PK (*n* = 3), or no information was available (*n* = 1). For KADCYLA, most of the patients were females (breast cancer). For race, the same pattern was noted as with sex. POPPK could not detect the impact of race on the PK for KADCYLA.

EMA [20] noted that “The adult population modelling showed that race did not significantly affect the PK of MYLOTARG (hP67.6 or unconjugated calicheamicin). Population PK modelling showed that race, in particular Asian versus non-Asian (White 89%, Black 2%, Other 2%, Asian 7%), was not a significant covariate on the pharmacokinetics of gemtuzumab ozogamicin”. It should be noted that the race-based sample size was not adequate enough to establish the impact of race on the PK of hP67.6 or un-conjugated calicheamicin in the POPPK study.

## 5. Pediatrics

So far, only two PK studies for ADCS have been conducted in the pediatrics and are summarized below (obtained from literature). The information from these studies were not included in the FDA package insert. For all 10 ADCs, the FDA package insert does not provide any PK information on the pediatrics.

### 5.1. MYLOTARG

The FDA package insert states that: “The safety and efficacy of MYLOTARG as a single agent in the pediatric patients with relapsed or refractory acute myeloid leukemia (AML) is supported by a single-arm trial in 29 patients in the following age groups: 1 patient 1 month to less than 2 years old, 13 patients 2 years to less than 12 years old, and 15 patients 12 years to 18 years old. A literature review included an additional 96 patients with ages ranging from 0.2 to 21 years. No differences in efficacy and safety were observed by age”.

The clinical pharmacology section under specific population states that age had no clinically significant effect on the PK of gemtuzumab ozogamicin. It is, however, not known whether the analysis included the pediatric data.

Buckwalter et al. [21] studied the PK of MYLOTARG in pediatric patients with relapsed or refractory AML. Plasma samples were analyzed for hP67.6 (antibody) and calicheamicin derivatives (total and un-conjugated) using validated enzyme-linked immunosorbent assay (ELISA) methods. The children age and weight ranged from 1 to 16 years and 9.9 to 65.1 kg (for 9 mg/m^2^) dose. There were two infants (0–2 years), five children (3–11 years), and seven adolescents (12–16 years). PK parameters were estimated by non-compartmental analysis. The clearance of MYLOTARG in infants, children, and adolescents was 30 mL/h, 60 mL/h, and 260 mL/h, respectively, compared to adult clearance of 270 mL/h. The volume of distribution at steady state (Vss) in infants, children, and adolescents was 2.9 L, 3.9 L, and 9.4 L, respectively, compared to adult Vss of 20 L. Although sample size was small, Buckwalter et al.’s study indicated that the PK of MYLOTARG is age-dependent in pediatric population. Both the clearance and volume of distribution of MYLOTARG increased with age, a general observation for both small and large molecules.

### 5.2. ADCETRIS

Flerlage et al. [19] conducted a POPPK study of ADCETRIS in 16 children (6–18 years of age) with Hodgkin lymphoma. There was 1 subject between 6–10 years of age, 4 subjects 11–15 years of age, and 11 subjects 16–18 years of age. The authors noted that the AUC and C_max_ of ADCETRIS were lower in pediatrics by 25% and 11%, respectively, as compared with adults. Most of the subjects were adolescents and it was not surprising that there was barely any difference in the AUC and C_max_ of ADCETRIS in this pediatric population from adults. Considering the age range in this study, it is anticipated that children under 12 years of age, especially below 5 years of age, may have substantially different PK than adults. However, it is also important to note that some medicines may not be given to young children due to the nature of the disease.

The greatest impact of age on the PK of a drug (small or large molecules) is on the younger age groups, generally ≤5 years of age. A general stratification of age groups should be ≤5 years (may be stratified within two age groups: ≤2 years and >2 years to 5 years), >5 years to 12 years and >12–<18 years of age [18,22].

## 6. Renal Impairment

The payload of ADCs are generally small molecules, and based on the experience with small molecules, it is anticipated that renal impairment for those small molecules that are renally excreted will have an impact on the PK (higher exposure or reduced clearance). With the exception of ADCETRIS, the impact of severe renal impairment was not studied for the other nine ADCs. Based on the FDA package inserts, POPPK analysis did not detect any impact of renal impairment on the PK of ADCs. It should be noted that it is severe renal impairment where the highest impact on the PK of an ADC or its payload may (highly likely) be observed.

**Table 3:** Impact of renal and hepatic impairment on the pharmacokinetics of ADCs.

ADCs	Renal Impairment (RI)	Hepatic Impairment (HI)
ZYNLONTA (loncastuximab tesirine-lpyl), 2021	The excretion pathways of SG3199 (small molecule) were not studied in humans and it was speculated that SG3199 will be minimally renally excreted. No impact of mild or moderate RI on the PK of ZYNLONTA was noted. The impact of severe RI and end-stage renal disease (ESRD) with or without hemodialysis on ZYNLONTA PK is not known.	Loncastuximab tesirine-lpyl (antibody) is expected to be metabolized into small peptides by catabolic pathways. The small molecule SG3199, is metabolized by CYP3A4/5 in vitro. Mild HI may increase the exposure of un-conjugated SG3199. The impact of moderate or severe HI on the PK of ZYNLONTA is not known.
BLENREP (belantamab mafodotin-blmf), 2020	Mild or moderate RI had no clinically significant impact on the PK of BLENREP. The impact of severe RI or ESRD not on dialysis or requiring dialysis on the PK of BLENREP is not known.	Mild HI had no clinically significant impact on the PK of BLENREP. The impact of moderate and severe HI on the PK of BLENREP is not known.
TRODELVY (sacituzumab govitecan-hziy), 2020	The small molecule of TRODELVY is SN-38 which is not eliminated renally. There are no data on the PK of TRODELVY in patients with renal impairment or end-stage renal disease	The PK of TRODELVY was similar between patients with normal hepatic function (*n* = 45) and with mild HI (*n* = 12) but is not known in patients with moderate and severe HI. SN-38 exposure may be elevated in patients with hepatic impairment due to decreased hepatic UGT1A1 activity [10].
ENHERTU (fam-trastuzumab deruxtecan-nxki), 2019	POPPK analysis indicated that the PK of ENHERTU is not influenced by mild (*n* = 206; normal = 238) or moderate RI (*n* = 57). No information is available in patients with severe RI.	POPPK analysis indicated that the PK of ENHERTU is not influenced by mild (*n* = 215; normal = 283) or moderate HI (*n* = 4). No information is available in patients with severe HI. In patients with moderate hepatic impairment, there is a possibility of increased exposure related to the topoisomerase inhibitor.
PADCEV (enfortumab vedotin-ejfv) 2019	Following 1.2 mg/kg dose of PADCEV, mild, moderate, and severe RI impairment had no impact on the PK of PADCEV or un-conjugated monomethyl auristatin E (MMAE). The effect of ESRD with or without dialysis on the PK of PADCEV or un-conjugated MMAE is not known	POPPK study indicated that there was a 48% increase in the AUC of un-conjugated MMAE in patients with mild HI as compared to subjects with normal hepatic function. The effect of moderate or severe HI on the PK of PADCEV or un-conjugated MMAE is not known.
POLIVY (polatuzumab vedotin-piiq) 2019	No difference in the PK of conjugated and un-conjugated MME was noted between patients with mild or moderate RI and normal renal function. The impact of severe RI and in patients with ESRD on the PK of MME is not known.	In patients with mild HI, the PK of monomethyl auristatin E (MME) was similar between patients with normal hepatic function but un-conjugated MME was higher by 40% in subjects with mild HI. The impact of moderate and severe hepatic impairment or liver transplantation on the PK of MME is not known.
BESPONSA (inotuzumab ozogamicin), 2017	Based on the POPPK analysis, the clearance of BESPONSA in patients with mild (*n* = 237), moderate (*n* = 122), and severe (*n* = 4) RI was similar to the clearance in patients with normal renal function (*n* = 402). The safety and efficacy of BESPONSA in patients with ESRD with or without hemodialysis is not known.	The clearance of BESPONSA in patients with mild (*n* = 150) HI was similar to patients with normal (*n* = 611) hepatic function. The impact of moderate and severe hepatic impairment on the PK of BESPONSA is not known
KADCYLA (ado-trastuzumab emtansine), 2013	POPPK analysis indicated that the PK of KADCYLA was not influenced by mild or moderate RI. No information is available in patients with severe RI.	The AUCs of KADCYLA after the first dose in patients with mild and moderate HI were approximately 38% and 67% lower than that of patients with normal hepatic function, respectively. KADCYLA has not been studied in patients with severe HI.
ADCETRIS (brentuximab vedotin), 2011 Baiting Zhao	The small molecule MMAE of ADCETRIS is renally excreted. Following 1.2 mg/kg dose of ADCETRIS, the PK of MMAE was evaluated in subjects with mild (*n* = 4), moderate (*n* = 3), and severe (*n* = 3) RI. Renal impairment had no impact on the PK of ADC. The AUC of MMAE was approximately twofold higher in patients with severe RI compared to patients with normal renal function. Mild and moderate RI had no impact on the PK of MMAE [23].	MMAE is also metabolized by the liver. Following 1.2 mg/kg dose of ADCETRIS, the PK of MMAE was evaluated in subjects with mild (*n* = 1), moderate (*n* = 5), and severe (*n* = 1) HI. HI had no impact on the PK of ADC. The AUC of MMAE in mild, moderate, and severe HI was 3.5, 2.2, and 1.77-fold higher, respectively, compared to patients with normal hepatic function [23].
MYLOTARG (gemtuzumab ozogamicin), 2000	Based on the POPPK analysis, the clearance of MYLOTARG in patients with mild (*n* = 149) and moderate RI (*n* = 47) was similar to the clearance of MYLOTARG in patients with normal renal function (*n* = 209). The impact of severe renal impairment is not known.	There was no impact of mild HI on the PK of MYLOTARG and the impact of moderate and severe HI on the PK of MYLOTARG is not known.

In a study [23], following 1.2 mg/kg dose of ADCETRIS, the PK of monomethyl auristatin E (MMAE), the small molecule of the ADC was evaluated in subjects with normal renal function (*n* = 8); and mild (*n* = 4), moderate (*n* = 3), and severe (*n* = 3) renal impairment.

The AUC of ADC was 7% lower, 22% higher, and 71% lower in subjects with mild, moderate, and severe renal impairment as compared with subjects with normal renal function. The AUC of MMAE was comparable in subjects with mild and moderate renal impairment with subjects with normal renal function. In subjects with severe renal impairment, the AUC of MMAE was almost twofold higher than the subjects with normal renal function. In this study, the sample size was small, but it still provided some insight about the impact of renal impairment on the antibody and the payload of ADCETRIS [23].

The FDA package insert indicates to “Avoid the use of ADCETRIS in patients with severe renal impairment”. Generally, based on the exposure or clearance of a drug in patients with renal impairment, the dose is adjusted. However, the dosing recommendation of ADCETRIS in patients with severe renal impairment is surprising (avoid the drug altogether in this population). Dose adjustment may be also difficult for an ADC, even if the exposure of its payload (generally small molecule) is substantially increased in a disease state since ADCs also contains a monoclonal antibody. It is the payload that is toxic to the tumors, and a substantial increase in the exposure of the payload may cause harm to the healthy cells. However, dose adjustment of an ADC should be considered if a substantial exposure of the payload of an ADC in patients with renal impairment is noted rather than avoiding the drug altogether in severe renal impairment, depriving the patients of the therapeutic benefit of an ADC.

There are two important points to consider for ADCs with respect to renal impairment. For small molecules that are renally excreted, the exposure is increased as a function of mild, moderate, or severe renal impairment as compared to subjects with normal renal function; in this situation, adjustment of dose of a small molecule is straightforward. For ADCs, it appears to be a different situation. For the mab portion of the ADCS, renal impairment leads to a decrease in the AUC as noted by Zhao et al. [23] in their study of ADCETRIS. Although the sample size was small, the study still provided some insight about the direction and magnitude of the impact of renal impairment on the mab portion of ADCETRIS. From this study, it appears that a decrease in exposure is highest in the subjects with severe renal impairment and the lowest in mild renal impairment. On the other hand, the AUC of the payload in ADCETRIS, which is a small molecule, increased substantially in subjects with severe renal impairment.

Therefore, for ADCS, dose adjustment in subjects with renal impairment is a dilemma. Should one ignore the change in the exposure of mab portion of an ADC and adjust the dose based on only the payload, which is supposed to be toxic to the tumors, or consider both of them for dose adjustment?

As with small molecules, for therapeutic proteins (antibodies and non-antibodies), the impact of renal impairment and end-stage renal disease (ESRD) on the PK and subsequently on protein’s efficacy and toxicity should be considered. Kidneys play an important role in the catabolism and elimination of therapeutic proteins. However, there is a size cutoff point (below 60 kDa) for therapeutic proteins to be eliminated by glomerular filtration [24]. Therefore, theoretically, one will anticipate that renal impairment will not have any impact on the PK of large proteins such as monoclonal antibodies, but one will observe the impact of renal impairment on the PK of smaller proteins and peptides below the cutoff point of 60 kDa, such as interleukin-10, growth hormone, erythropoietin, and anakinra [24].

In a study, Czok et al. [25] evaluated the impact of severe renal impairment and ESRD on the PK of peptides and proteins. Based on two-drug analysis, the authors noted a continuous non-linear relationship between molecular weight (1.02 to 150 kDa) and changes in exposure in patients with severe renal impairment and ESRD. The authors concluded that based on their analysis, relevant changes in the exposure in severe renal impairment and ESRD should be expected for drugs with a molecular weight below 50 kDa. It should be noted that three ADCs have peptide as a payload. These ADCS are BLENREP (belantamab mafodotin-blmf), PADCEV (enfortumab vedotin-ejfv), and POLIVY (polatuzumab vedotin-piiq).

Based on the published literature, it appears that renal impairment (at least moderate and severe) and ESRD may have a clinically meaningful impact on the PK on ADCs, especially in severe renal impairment, and this will require dose adjustment. Therefore, renal impairment studies must be conducted for ADCs, especially in severe renal impairment. Considering the unique nature of ADCs (a monoclonal antibody and a small molecule or a peptide as payload), more stringent evaluation of the impact of renal impairment is needed. One can initiate a renal impairment study in subjects with severe renal impairment, and based on the results, may proceed to conduct a study in subjects with moderate and/or mild impairment.

## 7. Hepatic Impairment

The small molecule payload of an ADC can be metabolized by cytochrome-p450 system and can be a transporter for P-glycoprotein (P-gp). The FDA package insert indicates that most of the studies were conducted in patients with mild hepatic impairment, and no difference was found between subjects with normal hepatic function and mild hepatic impairment. No data are available for subjects with severe hepatic impairment, with the exception of ADCETRIS (from the literature).

In a study [23], following 1.2 mg/kg dose of ADCETRIS, the PK of ADC and monomethyl auristatin E (MMAE), the small molecule of the ADC was evaluated in subjects with normal hepatic function (*n* = 8); and mild (*n* = 1), moderate (*n* = 5), and severe (*n* = 1) hepatic impairment.

The AUC of ADC decreased in subjects with hepatic impairment. The AUC was 57%, 65%, and 71% in subjects with mild, moderate, and severe hepatic impairment, respectively, as compared to subjects with normal hepatic function. The AUC of MMAE in mild, moderate, and severe hepatic impairment was 3.5, 2.2, and 1.77-fold higher, respectively, compared to subjects with normal hepatic function. In this study, the sample size was too small to make any definite conclusion, but it still provided some insight about the impact of hepatic impairment on the antibody and the payload. FDA package insert states to “Avoid the use of ADCETRIS in patients with moderate or severe hepatic impairment”.

As with renal impairment, dose adjustment of an ADC should be considered if a substantial exposure of the payload of an ADC is noted in patients with hepatic impairment rather than avoiding the drug altogether in moderate or severe hepatic impairment, depriving the patients from the therapeutic benefit of an ADC.

Two studies (2013 and 2019) from the FDA evaluated the impact of hepatic impairment on therapeutic proteins and ADCs.

Yang et al. [26] reviewed the impact of hepatic impairment of 91 therapeutic proteins (TPs) approved by the FDA (until 2013, based on the pharmaceutical companies’ submissions to the FDA). The TPs included in this survey were cytokines and growth factors (*n* = 23), enzymes (*n* = 23), monoclonal antibodies (*n* = 32), and others (*n* = 13). The authors noted that no dedicated PK study was conducted in patients with hepatic impairment. Based on POPK analysis for seven TPs, it was found that hepatic impairment had no impact on the PK of these TPs with the exception of 25% higher clearance (lower exposure) of drotrecogin alfa. The authors concluded that a dedicated PK study for TPs in patients with hepatic impairment was not necessary (it does not seem a logical conclusion based on the sample size (seven TPs)). However, the authors suggested that considering that the data were very limited, it was important to collect more PK data of TPs in patients with hepatic impairment.

In another study, Sun et al. [27] evaluated the impact of hepatic impairment on the PK of monoclonal antibodies (*n* = 20) as well as ADCs (*n* = 4) with a focus on the mAb component. The data were collected from the pharmaceutical companies’ submissions to the FDA from 2013 to 2018. The FDA survey indicated that there were almost no data for severe HI, limited data for moderate HI, and abundant data for mild HI. A significant decrease in AUC was found for several mAbs or ADCs, and a trend for decreasing AUC was observed for other mAbs. The authors found a decrease in AUC of KADCYLA (ado-trastuzumab emtansine), ADCETRIS (brentuximab vedotin), and MYLOTARG (gemtuzumab ozogamicin). The authors’ overall conclusions were that hepatic impairment might impact the elimination of the mabs as well as the mab portion of the ADCs. Several mechanisms might be involved in reducing the AUC of the mabs and the mab portion of ADCs. More data should be collected to evaluate the impact of hepatic impairment on the mabs as well as mab portion of the ADCs. However, the impact of hepatic impairment on the payload of ADCS must be evaluated since these are small molecules. Experience dictates that the impact of hepatic impairment on the small molecules that are extensively metabolized will be immense.

Like renal impairment, there are also two important points to consider with hepatic impairment. For small molecules that are metabolized, the exposure is increased as a function of mild, moderate, or severe hepatic impairment as compared to subjects with normal hepatic function. For ADCs, it appears to be a different situation. For the mab portion of the ADCS, hepatic impairment leads to a decrease in the AUC, as noted by Zhao et al. [23] in their study of ADCETRIS. Although the sample size was small, the study still provided some insight into the direction and magnitude of the impact of hepatic impairment on the mab portion of ADCETRIS. From this study, it appeared that the decrease in exposure was highest in the subjects with mild hepatic impairment and the lowest with severe hepatic impairment. A similar observation was noted by Sun et al. [27] in subjects with mild and moderate hepatic impairment. On the other hand, the AUC of the payload in ADCETRIS, which is a small molecule, increased with the severity of the hepatic impairment. This is in line with the observations for small molecules.

Therefore, for ADCS, dose adjustment in subjects with hepatic impairment such as renal impairment is also a dilemma. Should one ignore the change in the exposure of mab portion of an ADC and adjust the dose based on payload, which is supposed to be toxic to the tumors, or consider both of them for dose adjustment?

## 8. Drug Interaction Studies

With the exception of TRODELVY, drug interaction studies of ADCS were reasonably well conducted and described in the package inserts. For TRODELVY, no drug interaction study was conducted. If a dedicated drug interaction study was not carried out, then at least in vitro studies were conducted to determine the impact of metabolizing enzymes and transporters on the small molecule portion of the ADCs. Some speculative thoughts were presented in some cases, but these were based on the experience with other drugs. Since ADCs contain monoclonal antibodies, the interaction of antibodies with other drugs, especially with small molecules, cannot be ignored and must be conducted. Monoclonal antibodies can induce or inhibit the cytochrome P-450 system, which can impact the PK of small molecules. This is possible if an ADC is given with another monoclonal antibody or a small molecule. This has been comprehensively described in a review by Mahmood and Green [28].

**Table 4:** Drug Interaction studies of ADCs.

ADCs	Drug Interaction
ZYNLONTA (loncastuximab tesirine-lpyl), 2021	In Vitro Studies: Cytochrome P450 (CYP) Enzymes: SG3199 does not inhibit CYP1A2, CYP2A6, CYP2B6, CYP2C8, CYP2C9, CYP2C19, CYP2D6, CYP2E1, or CYP3A4/5 at clinically relevant un-conjugated SG3199 concentrations. Transporter Systems: SG3199 is a substrate of P-glycoprotein (P-gp), but not a substrate of breast cancer resistance protein (BCRP), organic anion-transporting polypeptide (OATP)1B1, or organic cation transporter (OCT)1. SG3199 does not inhibit P-gp, BCRP, OATP1B1, OATP1B3, organic anion transporter (OAT)1, OAT3, OCT2, OCT1, multi-antimicrobial extrusion protein (MATE)1, MATE2-K, or bile salt export pump (BSEP) at clinically relevant un-conjugated SG3199 concentrations
BLENREP (belantamab mafodotin-blmf), 2020	Monomethyl auristatin F (MMAF), a payload, is a substrate of organic anion transporting polypeptide (OATP)1B1 and OATP1B3, multidrug resistance-associated protein (MRP)1, MRP2, MRP3, bile salt export pump (BSEP), and a possible substrate of P-gp.
TRODELVY (sacituzumab govitecan-hziy), 2020	No drug–drug interaction studies were conducted with TRODELVY or its components. Inhibitors or inducers of UGT1A1 are expected to increase or decrease SN-38 exposure, respectively.
ENHERTU (fam-trastuzumab deruxtecan-nxki), 2019	Impact of CYP3A4 inhibitors (itraconazole), OATP inhibitors (retonavir) on the PK of ENHERTU was not clinically meaningful. ENHERTU does not inhibit CYP1A2, CYP2B6, CYP2C8, CYP2C9, CYP2C19, CYP2D6 and CYP3A nor induce CYP1A2, CYP2B6, or CYP3A. At clinically relevant concentrations, ENHERTU has a low potential to inhibit P-gp.
PADCEV (enfortumab vedotin-ejfv) 2019	Drug–drug interaction studies of PADCEV were not formally evaluated. Ketoconazole (a strong CYP3A4 inhibitor) increased MMAE C_max_ by 25% and AUC by 34%. rifampin (a strong CYP3A4 inducer) decreased MMAE C_max_ by 44% and AUC by 46%.
POLIVY (polatuzumab vedotin-piiq) 2019	No dedicated drug–drug interaction clinical study of POLIVY was conducted. POPPK analysis indicated that concomitant rituximab was associated with increased conjugated MMAE AUC by 24% and decreased un-conjugated MMAE AUC by 37%.
BESPONSA (inotuzumab ozogamicin), 2017	N-acetyl-gamma-calicheamicin dimethylhydrazide is a substrate of P-glycoprotein (P-gp). At clinically relevant concentrations, N-acetyl-gamma-calicheamicin dimethylhydrazide had a low potential to induce or inhibit cytochrome P450 enzymes, inhibit UGT enzymes and drug transporters. At clinically relevant concentrations, BESPONSA had a low potential to induce or inhibit cytochrome P450 enzymes.
KADCYLA (ado-trastuzumab emtansine), 2013	No formal clinical drug–drug interaction studies of KADCYLA were performed. T-DM1 (trastuzumab-MCC-DM1) is expected to undergo catabolism by means of proteolysis in cellular lysosomes, with no significant involvement of CYP enzymes. In vitro metabolism studies in human liver microsomes suggest that DM1, the cytotoxic component of KADCYLA, is metabolized mainly by CYP3A4 and, to a lesser extent, by CYP3A5.
ADCETRIS (brentuximab vedotin), 2011	MMAE is primarily metabolized by CYP3A. Co-administration of ADCETRIS with ketoconazole, a potent CYP3A4 inhibitor, increased exposure to MMAE by approximately 34%. Co-administration of ADCETRIS with rifampin, a potent CYP3A4 inducer, reduced exposure to MMAE by approximately 46%. Co-administration of ADCETRIS with P-gp inhibitors may increase exposure to MMAE.

## 9. Immunogenicity

From the FDA package inserts, it can be concluded that immunogenicity studies were conducted for at least nine ADCs (exception being MYLOTARG). For MYLOTARG, the assessment report by the European Medicine Agency [20] indicated that across the four clinical studies, the incidence rate of anti-drug antibodies (ADA) development following MYLOTARG treatment was <1%. From the FDA package inserts, it seems that the impact of anti-drug antibodies on the PK, efficacy, and safety remains unknown for all 10 approved ADCs. It was mentioned in the PI that the presence of anti-BESPONSA antibodies did not have impact on the clearance of BESPONSA. Overall, immunogenicity incidence for ADCs do not appear to be high. The incidence of neutralizing antibodies should be investigated and its impact on the PK, efficacy, and safety should be evaluated.

**Table 5:** Immunogenicity studies of ADCs.

ADCs	Immunogenicity
ZYNLONTA (loncastuximab tesirine-lpyl), 2021	No patient (*n* = 134) was tested positive for antibodies against ZYNLONTA after treatment. The impact of anti-drug antibodies to ZYNLONTA on the PK, efficacy, or safety is not known.
BLENREP (belantamab mafodotin-blmf), 2020	In clinical studies of BLENREP, two patients (*n* = 274) tested positive for anti-BLENREP antibodies after treatment.
TRODELVY (sacituzumab govitecan-hziy), 2020	Persistent anti- TRODELVY antibodies developed in two patients (*n* = 106).
ENHERTU (fam-trastuzumab deruxtecan-nxki), 2019	In four patients (*n* = 640), ENHERTU related anti-drug antibodies (ADA) were found. Neutralizing anti-ENHERTU antibodies were not assessed.
PADCEV (enfortumab vedotin-ejfv) 2019	Four patients (*n* = 365) were found to be transiently positive for anti-padcev antibody.
POLIVY (polatuzumab vedotin-piiq) 2019	Eight patients (*n* = 134) were tested positive for antibodies against POLIVY at one or more post-baseline time points.
BESPONSA (inotuzumab ozogamicin), 2017	Seven (*n* = 236) patients were tested positive for anti-BESPONSA antibodies and the presence of anti-BESPONSA antibodies did not have impact on the clearance of BESPONSA. Neutralizing antibodies were not detected in any patient.
KADCYLA (ado-trastuzumab emtansine), 2013	Following KADCYLA dosing, from seven clinical studies, 63 patients (*n* = 1243) patients tested positive for anti-KADCYLA antibodies at one or more post-dose time points. In two clinical studies, 39 patients (*n* = 867) were tested positive for anti-KADCYLA antibodies, of which 18 patients were tested positive for neutralizing antibodies.
ADCETRIS (brentuximab vedotin), 2011	Patients with Hodgkin lymphoma and Systemic anaplastic large cell lymphoma in the phase 2 trials were evaluated for antibodies to ADCETRIS every 3 weeks. Approximately 7% of patients in these trials developed persistently positive antibodies (positive test at more than two time points) and 30% developed transiently positive antibodies (positive in one or two post-baseline time points). Sixty-two percent of patients out of 58 with either transiently or persistently positive for ADCETRIS antibodies had neutralizing antibodies. The effect of anti-ADCETRIS antibodies on the safety, PK, and efficacy of ADCETRIS is not known
MYLOTARG (gemtuzumab ozogamicin), 2000	The US FDA package insert [11] mentioned that “Immunogenicity of MYLOTARG was not studied in clinical trials using the recommended dose regimens”. The assessment report by the European Medicine Agency [24] indicated that across the four clinical studies, the incidence rate of ADA development following MYLOTARG treatment was <1%.

## 10. Pregnancy

There is no dedicated evaluation of the impact of pregnancy on the PK, efficacy, and safety of the ADCs. The package inserts of ADCs provide a general statement: “Based on its mechanism of action, ADC X can cause embryo-fetal harm when administered to a pregnant woman, because it contains a genotoxic compound and affects actively dividing cells. There are no data on the use of ADC X in pregnant women to evaluate for drug-associated risk, major birth defects and miscarriage”. It should be noted that the information on embryo-fetal harm comes from rat studies (embryo-fetal development) at higher or equal to the human recommended dose. Overall, there is no definite information in the FDA package insert related to pregnancy and the use of ADCs in pregnant women.

It appears that the FDA uses a cautionary language for the use of ADCs in pregnant women but does not contraindicate. This is, however, a serious issue because a lot of pregnant women will be taking one of these ADCs without knowing its harmful effect to the fetus or to their own health. Studies must be conducted in this direction.

**Table 6:** ADCs in pregnancy and lactation.

ADCs	Pregnancy	Lactation
ZYNLONTA (loncastuximab tesirine-lpyl), 2021	Based on its mechanism of action, ZYNLONTA can cause embryo-fetal harm when administered to a pregnant woman, because it contains a genotoxic compound (SG3199) and affects actively dividing cells. There are no data on the use of ZYNLONTA in pregnant women to evaluate for drug-associated risk.	There are no data on the presence of loncastuximab tesirine-lpyl or SG3199 in human milk, the effects on the breastfed child, or milk production.
BLENREP (belantamab mafodotin-blmf), 2020	Based on its mechanism of action, BLENREP can cause fetal harm when administered to a pregnant woman, because it contains a genotoxic compound (the microtubule inhibitor, MMAF) and it targets actively dividing cells. There are no data on the use of BLENREP in pregnant women to evaluate for drug-associated risk.	There are no data on the presence of belantamab mafodotin-blmf in human milk or the effects on the breastfed child or milk production.
TRODELVY (sacituzumab govitecan-hziy), 2020	Based on its mechanism of action, TRODELVY can cause teratogenicity and/or embryo-fetal lethality when administered to a pregnant woman. TRODELVY contains a genotoxic component, SN-38, and is toxic to rapidly dividing cells. There are no data in pregnant women to evaluate drug-associated risk.	There are no data regarding the presence of sacituzumab govitecan-hziy or SN-38 in human milk, the effects on the breastfed child, or the effects on milk production.
ENHERTU (fam-trastuzumab deruxtecan-nxki), 2019	Based on its mechanism of action, ENHERTU can cause fetal harm when administered to a pregnant woman. Based on its mechanism of action, the topoisomerase inhibitor component of ENHERTU, DXd, can also cause embryo-fetal harm when administered to a pregnant woman because it is genotoxic and targets actively dividing cells.	There are no data regarding the presence of fam-trastuzumab deruxtecan-nxki in human milk, the effects on the breastfed child, or the effects on milk production.
PADCEV (enfortumab vedotin-ejfv) 2019	There are no data on the use of PADCEV in pregnant women to evaluate for drug-associated risk, major birth defects and miscarriage.	There are no data on the presence of PADCEV in human milk or the effects on the breastfed child or milk production.
POLIVY (polatuzumab vedotin-piiq) 2019	POLIVY can cause fetal harm when administered to a pregnant woman. There are no data on the use of PADCEV in pregnant women to evaluate for drug-associated risk, major birth defects and miscarriage.	There is no data on the presence of POLIVY in human milk or the effects on the breastfed child or milk production.
BESPONSA (inotuzumab ozogamicin), 2017	Based on its mechanism of action BESPONSA can cause embryo-fetal harm when administered to a pregnant woman. There are no data on BESPONSA use in pregnant women to evaluate drug-associated risk of major birth defects and miscarriage.	There are no data on the presence of inotuzumab ozogamicin or its metabolites in human milk, the effects on the breastfed infant, or the effects on milk production.
KADCYLA (ado-trastuzumab emtansine), 2013	KADCYLA can cause fetal harm when administered to a pregnant woman. There are no data on the use of KADCYLA in pregnant women. Based on its mechanism of action, the DM1 component of KADCYLA can also cause embryo-fetal harm when administered to a pregnant woman.	There is no information regarding the presence of ado-trastuzumab emtansine in human milk, the effects on the breastfed infant, or the effects on milk production.
ADCETRIS (brentuximab vedotin), 2011	There are no adequate and well-controlled studies with ADCETRIS in pregnant women. ADCETRIS can cause fetal harm when administered to a pregnant woman.	It is not known whether brentuximab vedotin is excreted in human milk.
MYLOTARG (gemtuzumab ozogamicin), 2000	Based on its mechanism of action, MYLOTARG can cause embryo-fetal harm when administered to a pregnant woman. There are no data on MYLOTARG use in pregnant women to evaluate a drug-associated risk of major birth defects and miscarriage.	There are no data on the presence of gemtuzumab ozogamicin or its metabolites in human milk, the effects on the breastfed infant, or the effects on milk production.

## 11. Lactation

There is no dedicated study about the appearance of ADCs or their metabolites in human milk. The package inserts of ADCs provide a general statement: “There are no data on the presence of ADC X in human milk, the effects on the breastfed child, or milk production”. For some ADCs, a time limit has been set for not breast feeding a child for at least several weeks or months after the last dose of an ADC taken by the mother. The FDA also emphasizes that “a decision should be made whether to discontinue nursing or to discontinue the drug, taking into account the importance of the drug to the mother”.

## 12. Conclusions

This review summarizes the intrinsic and extrinsic factors on the PK of ADCs. It is well established that intrinsic and extrinsic factors generally have a substantial impact on the PK of small molecules but the impact of these factors has not been comprehensively studied for macromolecules.

Since the ADCs are combination products of a large molecule (monoclonal antibody) and a small molecule (payload), it is very important that the impact of intrinsic and extrinsic factors on the PK of both these molecules be studied. From this review, it is evident that both intrinsic and extrinsic factors do have an impact on the PK of ADCs. However, the studies to detect the true impact of intrinsic and extrinsic factors on these molecules are not rigorous. For example, in a recent paper, Sun et al. [27] noted that the hepatic impairment may have impact on the PK of mAbs as well as ADCs. The authors noted that although there are abundant data for mild hepatic impairment, data in moderate and severe hepatic impairment are lacking. In a real world, it is moderate to severe hepatic impairment which will be the cause of concern. Similarly, limited data are available on the impact of renal impairment, especially on the PK of monoclonal antibodies [29]. It should be recognized that ADCs are linked with a small molecule and not to conduct renal or hepatic impairment studies for ADCs in moderate and severe renal and/or hepatic impairment due to the presence of small molecule is not scientifically rational. In particular, it is important to conduct such studies in patients with severe renal or hepatic impairment. The current ADCs’ package inserts of the FDA and EMA are lacking this very important information.

Drug–drug interaction studies are not only important for small molecules but also important for therapeutic proteins (both monoclonal antibodies and non-antibodies) [28]. Some small molecules may not have any impact on the PK of monoclonal antibodies, but antibodies can induce or inhibit the cytochrome P-450 system, which may lead to a significant impact on the PK of a small molecule [28]. Therefore, conducting drug–drug interaction studies to evaluate the impact of monoclonal antibodies on the small molecule when given with an ADC is very important and should be carried out with rigor.

Regarding the assessment of immunogenicity of ADCs, it is important to note the following statement in the US FDA package inserts of the ADCs: “Immunogenicity data are highly dependent on the sensitivity and specificity of the test methods used. Additionally, the observed incidence of a positive result in a test method may be influenced by several factors, including sample handling, timing of sample collection, drug interference, concomitant medication, and the underlying disease. Therefore, comparison of the incidence of antibodies to a product with the incidence of antibodies to other products may be misleading”. This statement is of practical value and should be considered during the development of ADCs and the interpretation of immunogenecity data. The immunogenicity studies were conducted for ADCs, but the impact of neutralizing antibodies on the PK, safety, and efficacy is missing.

Attempts were made to determine the impact of intrinsic and extrinsic factors on the PK of ADCs through population pharmacokinetics (POPPK). It should, however, be important to recognize that in order to detect the influence of covariates on PK parameters from POPPK, the sample size should be adequate (large enough to detect the influence of the covariate). It appears that in many cases such as age, gender, severe hepatic or renal impairment, and drug–drug interaction studies, the sample size was simply not enough to detect the impact of intrinsic and extrinsic factors on the PK of these ADCs. The ultimate conclusion was that the impact of these factors is either unknown or there were not enough data to draw any conclusion. It is not enough to simply mention that there are not enough data to draw any conclusion regarding the impact of intrinsic and extrinsic factors on the PK of ADCs; rigorous studies are needed in this direction. For example, the conclusion of EMA [20] based on data (White 89%, Black 2%, Other 2%, Asian 7%) and POPPK analysis of MYLOTARG that race, in particular Asian versus non-Asian, was not a significant covariate on the PK of gemtuzumab ozogamicin was incorrect because there are not enough data to make such a conclusion.

Model-based conclusions require good data and a model which can be considered to some extent reliable. The underlying assumption(s) for constructing a model is the most important factor since these assumptions will dictate the potential accuracy of a model. Making assumptions in a biological or physiological system is far more complex than a physical system because very little is known about the biological or physiological mechanisms in the living organisms. One should not ignore the fact that all models are wrong, but some are useful [30]. Models in a biological or physiological system at best are an approximation and the most simplistic form of describing the biological or physiological processes. The regulatory agencies must avoid the approval of medicines based on the models. Off course, models are useful during drug development and should be used to move forward.

There is no information available regarding the PK, safety, and efficacy in children in the package inserts of ADCs. The two pediatric PK studies described in this review were obtained from the literature and showed that the PK and dose of ADCs can be age-dependent. Off course, one does not need a pediatric study if it will not be used in pediatrics.

Obesity is on the rise both in adults and children round the globe, and in modern day drug development, obesity should be considered as an important covariate.

The impact of pregnancy on the PK of ADCs remains unknown. Similarly, the excretion of either monoclonal antibody or the small molecule of the ADCs in mother’s milk is not known. Both of these are important factors and should be rigorously pursued.

Some ADCs (blenrep, adcetris, padcev, trodelvy, enhertu, and zynlonta) were approved by the FDA conditionally with the statement that “This indication is approved under accelerated approval based on tumor response rate and duration of response. Continued approval for this indication may be contingent upon verification and description of clinical benefit in a confirmatory trial”. This may be a dangerous practice and only time will tell if this approach (conditional approval) of approving a drug for marketing is appropriate or not. It will be interesting to see if the regulatory agencies in other parts of the world will consider approving medicines in this way.

In short, ADCs are useful therapeutic agents for the treatment of cancer. However, the therapeutic benefits of ADCs can be extended to other indications such as inflammatory diseases and atherosclerosis. A more stringent approach for the development and approval of ADCs are needed. The impact of intrinsic and extrinsic factors should be evaluated rigorously so that an optimum dose can be selected for a patient or a patient population with certain background.

## Figures and Tables

**Table 1:** Description of ADCs.

ADCs	Description
ZYNLONTA (loncastuximab tesirine-lpyl) Initial U.S. Approval: 2021 [8]	ZYNLONTA is a CD19-directed antibody and alkylating agent conjugate indicated for the treatment of adult patients with relapsed or refractory large B-cell lymphoma. ZYNLONTA was approved under accelerated approval and the approval for this indication will continue after the clinical benefits are confirmed in confirmatory trial(s). ZYNLONTA is a humanized IgG1 kappa monoclonal antibody conjugated to SG3199 (small molecule cytotoxin), a pyrrolobenzodiazepine (PBD) dimer cytotoxic alkylating agent, through a protease-cleavable valine-alanine linker. SG3199 attached to the linker is designated as SG3249, also known as tesirine.
BLENREP (belantamab mafodotin-blmf) Initial U.S. Approval: 2020 [9]	Indicated for patients with relapsed or refractory multiple myeloma. Belantamab mafodotin-blmf is an antibody conjugate consisting of an afucosylated, humanized immunoglobulin G1 monoclonal antibody covalently linked to the microtubule inhibitor monomethyl auristatin F (MMAF) via a protease-resistant maleimidocaproyl linker. The small molecule component is MMAF, a microtubule inhibitor.
TRODELVY (sacituzumab govitecan-hziy) Initial U.S. Approval: 2020 [10]	Indicated for the treatment of adult patients with metastatic triple-negative breast cancer (mTNBC) who have received at least two prior therapies for metastatic disease. Sacituzumab govitecan-hziy (TRODELVY) is a trophoblast cell-surface antigen-2 (Trop-2) directed ADC, consisting of a humanized monoclonal antibody (sacituzumab), the small molecule drug SN-38 (a topoisomerase inhibitor), and a hydrolysable linker (called CL2A), which links the humanized monoclonal antibody to SN-38. SN-38 is an active metabolite of irinotecan and is formed via hydrolysis of irinotecan by carboxylesterases. SN-38 is metabolized via glucuronidation by UGT1A1.
ENHERTU (fam-trastuzumab deruxtecan-nxki) Initial U.S. Approval: 2019 [11]	Indicated for the treatment of adult patients with unresectable or metastatic HER2-positive breast cancer. ENHERTU is a HER2-directed ADC composed of a humanized anti-HER2 IgG1 monoclonal antibody covalently linked to a topoisomerase inhibitor via a tetrapeptide-based cleavable linker.
PADCEV (enfortumab vedotin-ejfv) Initial U.S. Approval: 2019 [12]	Indicated for the treatment of adult patients with locally advanced or metastatic urothelial cancer. Enfortumab vedotin-ejfv (PADCEV) is a Nectin-4 directed ADC comprised of a fully human anti-Nectin-4 IgG1 kappa monoclonal antibody (AGS-22C3) conjugated to the small molecule monomethyl auristatin E (MMAE) via a protease-cleavable maleimidocaproyl valine-citrulline (vc) linker (SGD-1006).
POLIVY (polatuzumab vedotin-piiq) Initial U.S. Approval: 2019 [13]	POLIVY in combination with bendamustine and a rituximab is indicated for the treatment of adult patients with relapsed or refractory diffuse large B-cell lymphoma. Polatuzumab vedotin-piiq (POLIVY) is a CD79b-directed ADC consisting of a humanized immunoglobulin IgG1 monoclonal antibody, the small molecule anti-mitotic agent MMAE, and a protease-cleavable linker maleimidocaproyl-valine-citrulline-p-aminobenzyloxycarbonyl (mc-vc-PAB) that covalently attaches MMAE to the polatuzumab antibody.
BESPONSA (inotuzumab ozogamicin) Initial U.S. Approval: 2017 [14]	BESPONSA is indicated for the treatment of adults with relapsed or refractory B-cell precursor acute lymphoblastic leukemia (ALL). Inotuzumab ozogamicin (BESPONSA) is a CD22-directed ADC consisting of recombinant humanized immunoglobulin IgG4 kappa antibody inotuzumab, specific for human CD22, a cytotoxic agent N-acetyl-gamma-calicheamicin dimethylhydrazide (small molecule), and an acid-cleavable linker composed of the condensation
BESPONSA (inotuzumab ozogamicin) Initial U.S. Approval: 2017 [14]	Product of 4-(4′-acetylphenoxy)-butanoic acid (AcBut) and 3-methyl-3-mercaptobutane hydrazide (known as dimethylhydrazide) that covalently attaches N-acetyl-gamma-calicheamicin to inotuzumab.
KADCYLA (ado-trastuzumab emtansine), Initial U.S. Approval: 2013 [15]	Indicated as a single agent, for the treatment of patients with HER2-positive, metastatic breast cancer who previously received trastuzumab and a taxane, separately or in combination [18]. KADCYLA (ado-trastuzumab emtansine) is an ADC consisting of a humanized anti-HER2 IgG1, (trastuzumab), covalently linked to the microtubule inhibitory drug DM1 (a microtubule-inhibitory maytansinoid) linked through thioether linker MCC (4-N-maleimidomethyl cyclohexane-1-carboxylate). Emtansine is the MCC-DM1 complex. DM1 and MCC are small molecules.
ADCETRIS (brentuximab vedotin) Initial U.S. Approval: 2011 [16]	Indicated for Hodgkin lymphoma and Systemic anaplastic large cell lymphoma. ADCETRIS (brentuximab vedotin) is a CD30-directed antibody–drug conjugate (ADC) consisting of chimeric IgG1 antibody cAC10, specific for human CD30, the microtubule disrupting agent monomethyl auristatin E (MMAE), and a protease-cleavable linker that covalently attaches MMAE to cAC10. The small molecule component is MMAE, a microtubule inhibitor.
MYLOTARG (gemtuzumab ozogamicin) Initial U.S. Approval: 2000 and then 2017 [17]	MYLOTARG was withdrawn from the market in 2010 due to adverse events particularly hepatic side effects. It was then approved in 2017. For the treatment of newly diagnosed CD33-positive acute myeloid leukemia (AML) in adults and the treatment of relapsed or refractory CD33-positive AML in adults and in pediatric patients 2 years and older. MYLOTARG is an ADC that consists of a monoclonal antibody (hP67.6; recombinant humanized immunoglobulin G4), covalently linked to the cytotoxic agent N-acetyl gamma calicheamicin (small molecule) through an AcBut (4-(4-acetylphenoxy) butanoic acid) linker.

## References

[1] Lambert J.M., Charles Q., Morris C.Q. (2017). Antibody-Drug Conjugates (ADCs) for Personalized Treatment of Solid Tumors: A Review. Adv. Ther..

[2] Liu R., Wang R.E., Wang F. (2016). Antibody-drug conjugates for non-oncological indications. Expert Opin. Biol. Ther..

[3] Deslandes A. (2014). Comparative clinical pharmacokinetics of antibodydrug conjugates in first-in-human Phase 1 studies. MAbs.

[4] Atkinson A.J., Atkinson A.J., Daniels C.E., Dedrick R.L., Grudzinskas C.V., Markey S.P. (2001). Introduction to Clinical Pharmacology. Principles of Clinical Pharmacology.

[5] Wagner J.G. (1968). Kinetics of Pharmacologic response, I. Proposed relationship between response and drug concentration in the intact animal and man. J. Theor. Biol..

[6] Tabrizi M., Bornstein G.G., Suria H. (2010). Biodistribution mechanisms of therapeutic monoclonal antibodies in health and disease. AAPS J..

[7] Mahmood I. (2021). Clinical Pharmacology of Antibody-Drug Conjugates. Antibodies.

[8] FDA Package Insert ZYNLONTA (Loncastuximab Tesirine-Lpyl). https://www.accessdata.fda.gov/drugsatfda_docs/label/2021/761196s000lbl.pdf.

[9] FDA Package Insert BLENREP (Belantamab Mafodotin-Blmf). https://www.accessdata.fda.gov/drugsatfda_docs/label/2020/761158s000lbl.pdf.

[10] FDA Package Insert TRODELVY (Sacituzumab Govitecan-Hziy). https://www.accessdata.fda.gov/drugsatfda_docs/label/2020/761115s000lbl.pdf.

[11] FDA Package Insert ENHERTU (Fam-Trastuzumab Deruxtecan-Nxki). https://www.accessdata.fda.gov/drugsatfda_docs/label/2019/761139s000lbl.pdf.

[12] FDA Package Insert PADCEV (Enfortumab Vedotin-Ejfv). https://www.accessdata.fda.gov/drugsatfda_docs/label/2019/761137s000lbl.pdf.

[13] FDA Package Insert POLIVY (Polatuzumab Vedotin-Piiq). https://www.accessdata.fda.gov/drugsatfda_docs/label/2019/761121s000lbl.pdf.

[14] FDA Package Insert BESPONSA (Inotuzumab Ozogamicin). https://www.accessdata.fda.gov/drugsatfda_docs/label/2017/761040s000lbl.pdf.

[15] FDA Package Insert KADCYLA (Ado-Trastuzumab Emtansine). https://www.accessdata.fda.gov/drugsatfda_docs/label/2019/125427s105lbl.pdf.

[16] FDA Package Insert ADCETRIS (Brentuximab Vedotin). https://www.accessdata.fda.gov/drugsatfda_docs/label/2014/125388_s056s078lbl.pdf.

[17] FDA Package Insert MYLOTARG (Gemtuzumab Ozogamicin). https://www.accessdata.fda.gov/drugsatfda_docs/label/2017/761060lbl.pdf.

[18] Mahmood I. (2016). Pharmacokinetic considerations in designing pediatric studies of proteins, antibodies, and plasma-derived products. Am. J. Ther..

[19] Flerlage J., Metzger M., Wu J., Panetta J.C. (2016). Pharmacokinetics, Immunogenicity, and Safety of Weekly Dosing of Brentuximab in Pediatric Patients with Hodgkin Lymphoma. Cancer Chemother. Pharmacol..

[20] (2018). Assessment Report Mylotarg (International Non-Proprietary Name: Gemtuzumab Ozogamicin).

[21] Buckwalter M., Dowell J.A., Kroth-Bradley J., Gorovits B., Mayer P.R. (2004). Pharmacokinetics of Gemtuzumab Ozogamicin as a Single-Agent Treatment of Pediatric Patients With Refractory or Relapsed Acute Myeloid Leukemia. J. Clin. Pharm..

[22] Mahmood I. (2014). Dosing in children: A critical review of the pharmacokinetic allometric scaling and modelling approaches in pediatric drug development and clinical settings. Clin. Pharmacokinet..

[23] Zhao B., Chen R., O’Connor O.A., Gopal A.K., Ramchandren R., Goy A., Matous J.V., Fasanmade A.A., Manley T.J., Han T.H. (2016). Brentuximab vedotin, an antibody-drug conjugate, in patients with CD30-positive haematologic malignancies and hepatic or renal impairment. Br. J. Clin. Pharmacol..

[24] Meibohm B., Zhou H. (2012). Characterizing the Impact of Renal Impairment on the Clinical Pharmacology of Biologics. J. Clin. Pharmacol..

[25] Czock D., Keller F., Seidling H.M. (2012). Pharmacokinetic predictions for patients with renal impairment: Focus on peptides and protein drugs. Br. J. Clin. Pharmacol..

[26] Yang Y., Shord S., Zhao H., Men Y., Rahman A. (2013). Are Hepatic Impairment Studies Necessary for Therapeutic Proteins?. Clin. Ther..

[27] Sun Q., Seo S., Zvada S., Liu C., Reynolds K. (2020). Does Hepatic Impairment Affect the Exposure of Monoclonal Antibodies?. Clin. Pharmacol. Ther..

[28] Mahmood I., Green M.D. (2007). Drug interaction studies of therapeutic proteins or monoclonal antibodies. J. Clin. Pharmacol..

[29] Lee E., Gibbs J.P., Emery M.G., Block G., Wasserman S.M., Hamilton L., Kasichayanula S., Hanafin P., Somaratne R., Egbuna O. (2019). Influence of Renal Function on Evolocumab Exposure, Pharmacodynamics, and Safety. Clin. Pharmacol. Drug Dev..

[30] Box G.E.P. (1976). Science and statistics. J. Am. Stat. Assoc..

